# Enhancement of DUSP14 (dual specificity phosphatase 14) limits osteoarthritis progression by alleviating chondrocyte injury, inflammation and metabolic homeostasis

**DOI:** 10.1080/21655979.2021.1979355

**Published:** 2021-10-04

**Authors:** Zandong Zhao, Jie Yang, Liang zhang, Yunping Zhou

**Affiliations:** a Department of Sports Medicine, Honghui Hospital, Xi’an Jiaotong University Health Science Center Xi’an, Shaanxi Province, China; b Department of Foot and Ankle Surgery, Honghui Hospital, Xi’an Jiaotong University Health Science Center Xi’an, Shaanxi Province, China; c Department of Hand Surgery, Honghui Hospital, Xi’an Jiaotong University Health Science Center Xi’an, Shaanxi Province, China

**Keywords:** Osteoarthritis, chondrocyte, inflammatory injury, metabolism homeostasis, dusp14

## Abstract

Osteoarthritis (OA) is a proverbial inflammatory degenerative joint disease associated with the acceleration of the aging process and is characterized by chondrocyte injury and articular cartilage degradation. Dual-specificity phosphatase 14 (Dusp14), a common member of the DUSP family, has been implicated in multiple inflammatory diseases and bone loss. Nevertheless, the function of DUSP14 in OA remains unclear. In the present study, down-regulation of DUSP14 was corroborated in anterior cruciate ligament transection (ACLT)-induced OA rats and interleukin-1β (IL-1β)-stimulated chondrocytes. Additionally, the gain of DUSP14 reversed IL-1β-induced inhibition of chondrocyte viability but attenuated cell apoptosis. Concomitantly, DUSP14 overexpression muted IL-1β-induced release of pro-inflammatory mediators NO and prostaglandin E2 (PGE2), as well as pro-inflammatory cytokine levels (IL-6 and TNF-α). Furthermore, up-regulation of DUSP14 overturned the effects of IL-1β on the inhibition of collagen II and aggrecan expression, and enhancement of A Disintegrin and Metalloproteinase with Thrombospondin Motifs 5 (ADAMTS5) and matrix metalloproteinases (MMPs; MMP3 and MMP-13). Mechanistically, DUSP14 elevation increased the p-Adenosine 5ʹ-monophosphate-activated protein activated protein kinase(AMPK), inhibitor of NF-κB (IκB) expression and decreased p-p65 NF-κB expression, indicating that DUSP14 might restore the AMPK-IκB pathway to restrain NF-κB signaling under IL-1β exposure. Notably, blockage of AMPK signaling muted the protective efficacy of DUSP14 elevation against IL-1β-induced inflammatory injury and metabolism disturbance in chondrocytes. Interestingly, histological evaluation substantiated that DUSP14 injection alleviated cartilage degradation in OA rats. Together, DUSP14 may ameliorate OA progression by affecting chondrocyte injury, inflammatory response and cartilage metabolism homeostasis, implying a promising therapeutic strategy against OA.

## Introduction

Osteoarthritis (OA) is a common whole-point and debilitating disease worldwide and is a major source of pain and disability among people. Epidemiological investigation predicts a higher prevalence of OA over the coming decades due to the aging and increasingly obese population worldwide [1]. By 2030, OA is expected to disable approximately 54 million people [2]. Currently, OA, as a debilitative and painful disease, has become a significant societal cost and global burden, especially for older adults [3]. Although advanced in the past few years, the current pharmacological options for OA have limited analgesic efficacy and undesirable side effects [4]. Additionally, although eventual joint replacement for OA patients can restore joint function and relieve pain, postoperative complications greatly restricts the application of this therapeutic strategy [5]. Therefore, the exploration and development of a new promising approach for OA are urgently needed.

OA is generally characterized by articular cartilage degeneration, chondrocyte apoptosis and intra-articular inflammation [1,6,7]. Usually, cartilage extracellular matrix (ECM) maintains metabolic homeostasis depending solely on the function of its resident cells, chondrocytes [7]. The dense ECM mainly comprises type II collagen (COL2A1) and aggrecan that support elastic response to pressure and shear stress as joints move. During the pathogenic progression of OA, chondrocytes tilt toward aberrant catabolism by decreasing collagen II and aggrecan expression and increasing catabolic enzyme matrix metalloproteinases (MMPs) levels, ultimately leading to articular cartilage destruction and degeneration [8,9]. Abundant work in this field also implicates inflammation in the initiation and development of OA pathogenesis. Interleukin-1β (IL-1β) is a key inflammatory cytokine that activates the nuclear factor kappa-light-chain-enhancer of activated B cells (NF-κB) signaling, which in turn aggravates the inflammatory response [6]. Additionally, as a key OA-contributing factor, abnormal activation of NF-κB results in chondrocyte injury, synovial inflammation and MMP production to induce articular joint destruction [10,11]. Therefore, NF-κB activation may lead to OA onset and progression, and has become a potential target for the therapeutic intervention for OA [11].

Dual-specificity phosphatase 14 (Dusp14) is a common member of the DUSP family and contains a conserved C-terminal catalytic domain that supports its phosphatase function. DUSP14 is widely accepted to phosphorylate various signaling pathways (eg. NF-kB) to participate multiple physiological processes, such as cellular apoptosis, tissue injury and oxidative stress. Noticeably, recent study has implicated DUSP14 in inflammation-related diseases [12–14]. For instance, DUSP14 exerts protective effects against ischemia/reperfusion (I/R) injury by suppressing inflammation and cell apoptosis [12,15]. In addition to its inhibitory effects on the inflammatory response, DUSP14 is required for maintaining metabolic homeostasis in liver [13]. Importantly, emerging evidence confirms that DUSP14 alleviates osteoclast generation and bone loss, indicating a critical role in bone disorder and osteoporosis [16]. Nevertheless, the function of DUSP14 in OA remains elusive.

Herein, we hypothesized that DUSP14 might exert the critical role in the development of OA. Therefore, in the present study, we sought to investigate the therapeutic effects of DUSP14 in OA rats. Additionally, we carried out a pilot experiment to test the roles and mechanism of DUSP14 in chondrocyte dysfunction under OA conditions. Our findings confirmed that DUSP14 elevation might alleviate IL-1β-induced chondrocyte dysfunction and the progression of OA in rats by regulating the AMPK signaling, supporting a promising therapeutic strategy for OA.

## Materials and methods

### Animal ethics statement

Male Sprague−Dawley (SD) rats with an average weight of 200–220 g were obtained from the Animal Laboratory Center of the Fourth Military Medical University. All animals were acclimatized for one week under standard conditions with normal lighting (12/12 h light-dark cycle) at a constant temperature of 23 ± 2°C and 60–70% humidity. Rats were allowed to raise food and water *ad libitum*. All experimental protocols were conducted following the National Institutes of Health (NIH) Guide for the Care and Use of Laboratory Animals, and ethically approved by the Institutional Animal Care and Use Committee of Xi’an Jiaotong University Health Science Center, Honghui Hospital (No. SYXK (Shan) 2020–006)

## Animal experiments and administration

Rats were randomly divided into three groups (n = 10 per group): control groups (also known as sham groups), OA groups with anterior cruciate ligament transection (ACLT) surgery, OA and lentivirus vector LV-DUSP14 groups that were injected with lentivirus vector LV-DUSP14 (GenePharma, Shanghai, China) as previously described [17]. After acclimation for one week, ACLT was performed in right knee joint to construct the OA animal model as previously reported [18]. Briefly, SD rats were anesthetized and temporarily fixed on a splint. Then, right knee joint areas of rats were sterilized and skin incision was made on the medial side of patellar tendon. The anterior cruciate ligament was subsequently transected to induce OA. Sham operation was performed by opening the joint capsule but without ACLT in the right knee of independent rats. For the therapeutic experiments, lentivirus vector LV-DUSP14 (1 × 10^9^ plaque-forming units (PFUs) in a total volume of 20 μl) was injected into knee at 48 h after surgery and every 2 weeks thereafter. Approximately 20 weeks later, rats were anesthetized and sacrificed by cervical dislocation without suffering anything, and the articular cartilage was collected for subsequent experiments.

## Histological observations

The collected cartilage of knee joint was fixed and immersed in 10% neutral buffered formalin at 4°C for 48 h. Subsequently, the samples were decalcified in 10% EDTA for two weeks. After that, the specimens were dehydrated in a graded ethanol series and embedded in paraffin. Histological changes were assessed after cutting into serial 5-μm thick sections and staining them with hematoxylin and eosin (H&E) and safranin O-fast green. Furthermore, the Osteoarthritis Research Society International (OARSI) scoring system was applied to assess cartilage degeneration [19].

## Isolation and culture of primary chondrocytes

Primary chondrocytes were isolated from rat articular cartilage according to a previous report [20]. Briefly, articular cartilage was collected, cut and digested with trypsin for 0.5 h. Then, the specimens were plated in collagenase II in DMEM medium at 37°C, and then centrifuged for 5 min. After that, the collected cells were cultured in DMEM medium supplemented with 10% fetal calf serum (FCS) under 5% CO2 at 37°C. The prepared primary chondrocytes were passed at 80% confluence and cells between passages 1 to 3 were enrolled for subsequent experiments. All cells were housed in a humidified atmosphere containing 5% CO2 at 37°C.

## Cell treatments and lentivirus vector infection

Chondrocytes were exposed to IL-1β (10 ng/ml) for 24 h to simulate OA conditions *in vitro*. Moreover, chondrocytes were infected with the lentivirus vector LV-DUSP14 and LV-control plasmids (GenePharma) at a confluence of 60% to induce DUSP14 overexpression. Approximately 48 h later, the efficacy on DUSP14 protein expression was analyzed by western blotting.

## Si-AMPK transfection

Chondrocytes were seeded into 24-well plates and grown to 60%-70% confluence. Then, cells were transfected with 50 nM of si-AMPK or scrambled siRNA (negative control, si-NC) (GenePharma) using Lipofectamine 2000 (Invitrogen, Carlsbad, CA). Fourteen hours after si-RNA treatment, the protein levels of AMPK were measured by western blotting.

## QRT-PCR assay

Articular cartilage was collected and homogenized. Then, total RNA from cartilage tissues and chondrocytes was extracted using the TRIzol reagent (Invitrogen). Next, cDNA was synthesized using the SuperScript II First-Strand Synthesis System (Invitrogen). The real-time PCR was then conducted to quantify the mRNA levels of DUSP14, MMP3, MMP-13, ADAMTS5, collagen II and aggrecan, as previously described [13]. All specific primer sequences for these genes were designed and obtained from Shenggong Biotechnology Co., Ltd. (Shanghai, China). Finally, all specimens were subjected to the ABI PRISM 7000 sequence detection system (Applied Biosystems), and the transcriptional levels of these genes were calculated using a quantified formula of 2^−ΔΔCt^ against GAPDH levels.

## Western blotting analysis

Protein lysates were prepared using RIPA buffer from articular cartilage tissues and chondrocytes treated with IL-1β, LV-DUSP14, or si-AMPK. The BCA protein assay kit (Pierce, Rockford, USA) was used to quantify protein concentration. Then, the extracted proteins (25 μg) were subjected to 12% SDS-PAGE and subsequently transferred onto nitrocellulose membranes. Subsequently, 5% nonfat milk was added to interdict the nonspecific binding in membranes. After rinsing with TBST, the membranes were incubated with specific primary antibodies against rat DUSP14 (#PA5-75,585; Invitrogen), aggrecan (#PA1-1746; Invitrogen), ADAMTS5 (#ab41037), collagen II (#ab188570), p-p65 NF-κB (phospho S536; #ab239882) and p65 NF-κB (#ab19870) (all from Abcam, Cambridge, MA, USA), IκB-α (#4812; Cell Signaling Technology, Beverly, MA, USA), AMPK (#5831; Cell Signaling Technology), and p-AMPK (phospho Thr172; #2535S; Cell Signaling Technology), and then were treated with horseradish peroxidase-conjugated secondary antibody. Ultimately, the binding signals were visualized by incubation with the ECL reagent (Beyotime, Shanghai, China). Image J software (Bethesda, MD, USA) was used to quantify the immunoreactive bands.

## Cell viability analysis by MTT

After grown to 70%-80% confluence, cells were stimulated with the indicated conditions. Then, cells were further incubated with medium containing 10 μl of MTT solution (#C0009S; Beyotime) for 4 h. After that, formatted formazan precipitate was solubilized by incubating with 100 μl of Formazan solution for 4 h. Finally, all samples were analyzed using a microplate reader (Bio-Rad, Hercules, CA, USA) to analyze cell viability by measuring the absorbance at 570 nm.

## Flow cytometry assay

Following different treatments, the commercial Annexin V-FITC apoptosis analysis Kit (#C1062S; Beyotime) was applied to assess cell apoptosis. Briefly, chondrocytes were collected and re-suspended in a binding buffer. Subsequently, 5 μl Annexin V-FITC and 10 μl PI were added for further incubation under dark for 20 min. Specimens were then subjected to a fow cytometer (BD Biosciences, San Jose, CA, USA) to discriminate cell apoptosis.

## Determination of inflammatory cytokines and MMP levels

Chondrocytes were pre-treated with LV-DUSP14, si-AMPK prior to IL-1β stimulation. After collection, the concentration of NO was evaluated using an NO detection kit (#A013-2-1; Nanjing Jiancheng Bioengineering Institute, Nanjing, China) based on the Griess reaction. The contents of inflammatory mediators PGE2, IL-6, TNF-α and MMP-3, and MMP-13 in the culture medium were quantified using the commercial ELISA kits (eBioscience, San Diego, CA, USA). All protocols were conducted according to the manufacturer’ instructions.

## Statistical analysis

All experiments were performed in triplicate and repeated at least three times. Data are represented as mean ± SD. The statistical comparisons were carried out via SPSS 19.0 software (SPSS Inc. Chicago, IL, USA). Differences between groups were analyzed by Student’s *t*-test (for two groups) and ANOVA with Student-Newman-Keuls post-hoc tests (for three or more groups). Statistical significance was set as P < 0.05.

## Results

### *The expression of DUSP14 decreased in the OA model* in vivo *and* in vitro

**Figure 1.**
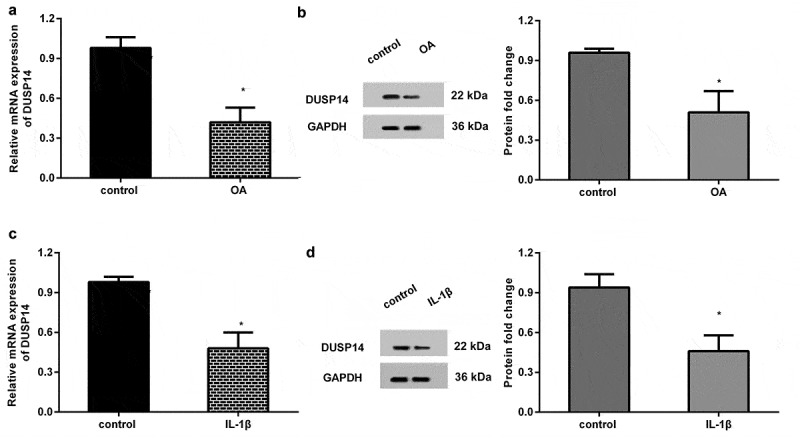
Downregulation of DUSP14 was confirmed in OA rats and IL-1β-stimulated chondrocytes. (a, b) The articular cartilage tissues were collected from ACLT-induced OA rats. Then, the mRNA (a) and protein (b) levels of DUSP14 were determined by qRT-PCR and western blotting. (c, d) Chondrocytes were treated with IL-1β for 24 h. Then, the mRNA (c) and protein (d) expression of DUSP14 were analyzed. n = 3. *P < 0.05 vs. control group

To ascertain the roles of DUSP14 in OA, rats underwent ACLT surgery to simulate OA progression. As shown in Figure 1a and 1b, the mRNA and protein levels of DUSP14 in knee articular cartilage were down-regulated in OA groups relative to those in the control groups. Simultaneously, the isolated chondrocytes stimulated with IL-1β exhibited lower transcript (Figure 1c) and protein (Figure 1d) levels of DUSP 14 than those from control groups. These data indicate the potential function of DUSP14 in the progression of OA.

## Elevation of DUSP14 alleviated IL-1β-evoked chondrocyte injury and inflammatory response

**Figure 2.**
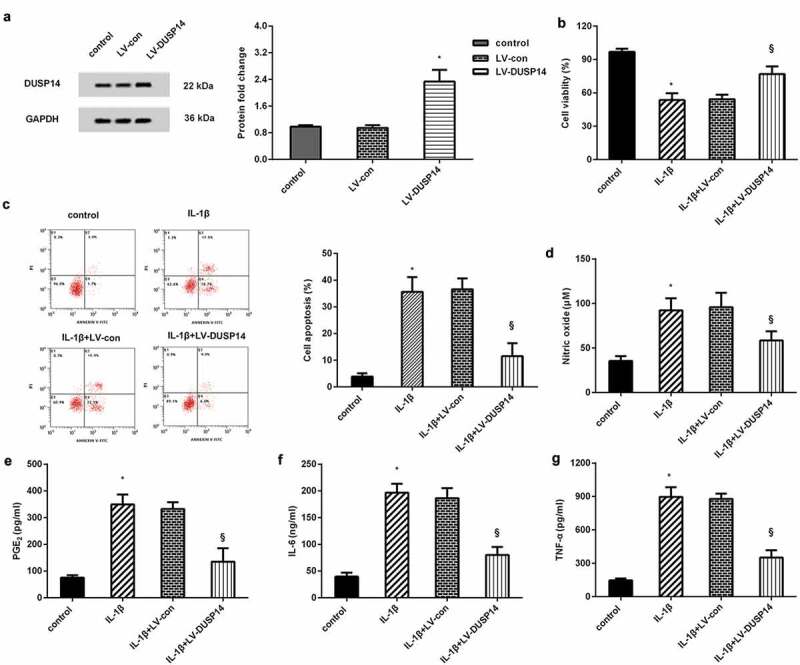
Elevation of DUSP14 antagonized IL-1β-evoked inflammatory injury in chondrocytes. (a) The effects of LV-DUSP infection were evaluated by western blotting. n = 3. (b, c) Before exposure to IL-1β, chondrocytes were treated with LV-DUSP14 vectors. Then, cell viability (b) and apoptosis (c) were analyzed by MTT and flow cytometer. n = 3. (d-g) The levels of inflammatory mediator NO and PGE2 and pro-inflammatory cytokines (IL-6 and TNF-α) were determined by the commercial kits. n = 4. *P < 0.05 vs. control group. ^§^P < 0.05 vs. IL-1β-treated group

In order to elaborate the function of DUSP14 in osteoarthritic chondrocyte function, the expression of DUSP14 was enhanced after LV-DUSP14 infection (Figure 2a). In contrast to the control groups, IL-1β treatment suppressed chondrocyte viability. Nevertheless, overexpression of DUSP14 reversed the inhibitory effects of IL-1β on cell viability (Figure 2b). Moreover, flow cytometry analysis corroborated stimulation with IL-1β induced chondrocyte apoptosis, which was restrained after DUSP14 enhancement (Figure 2c).

We next investigated DUSP14 function in IL-1β-induced chondrocyte inflammation, and the results confirmed that compared with control groups, higher inflammatory levels of mediator NO (Figure 2d) and PGE2 (Figure 2e) were determined in IL-1β-treated groups. Noticeably, these increases were abrogated after DUSP14 overexpression (Figure 2d and 2e). Additionally, chondrocytes exposed to IL-1β showed higher pro-inflammatory cytokine production (IL-6 and TNF-α) than the control groups. However, overexpression of DUSP14 attenuated IL-1β-induced release of IL-6 and TNF-α (figure 2f and 2g).

## DUSP14 affects metabolic homeostasis in IL-1β-stimulated chondrocytes

**Figure 3.**
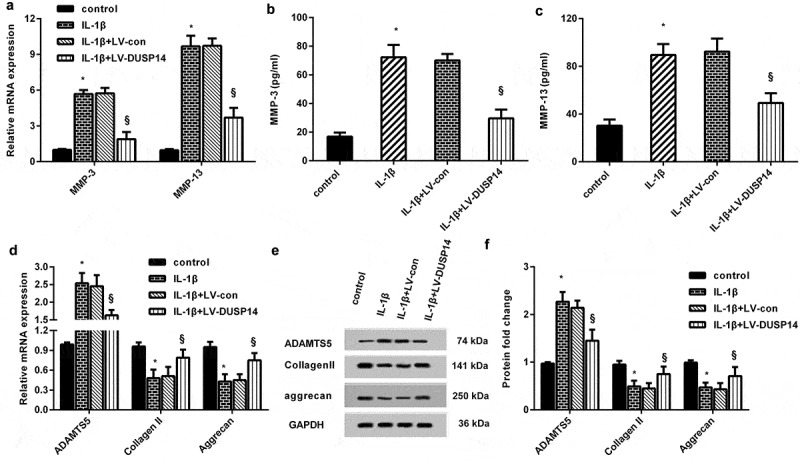
DUSP14 affected metabolic disorders in IL-1β-treated chondrocytes. (a) Cells were treated with LV-DUSP14 prior to IL-1β stimulation. Then, the mRNA levels of MMP-3 and MMP-13 were detected by qRT-PCR. n = 4. (b, c) The releases of MMP-3 (b) and MMP-13 (c) from chondrocytes were analyzed by ELSIA kits. n = 4. (d-f) The effects of DUSP14 overexpression on the mRNA (d) and protein expression (e, f) of ADAMTS5, collagen II and aggrecan were further evaluated. n = 4. *P < 0.05 vs. control group. ^§^P < 0.05 vs. IL-1β-treated group

The imbalance between catabolism and anabolism in chondrocytes is widely accepted as a key pathogenic factor in the progression of OA [9]. Therefore, we further investigated the function of DUSP14 in chondrocyte metabolic homeostasis under OA conditions *in vitro*. As shown in Figure 3a, IL-1β exposure enhanced the transcript of matrix catabolic MMP-3 and MMP-13. Concomitantly, the production of MMP-3 (Figure 3b) and MMP-13 (Figure 3c) was also elevated when chondrocytes were stimulated with IL-1β. Noticeably, DUSP14 overexpression mitigated IL-1β-induced these increases (Figure 3a-3c). Moreover, IL-1β treatment also elevated ADAMTS5 mRNA and protein levels, but reduced cartilage extracellular matrix anabolic component collagen II and aggrecan expression (Figure 3d-3f). These findings suggest that DUSP14 elevation may counteract IL-1β-evoked metabolic disturbance toward catabolism by regulating MMP and anabolism-related element expression.

## DUSP14 up-regulation regulates the AMPK/IκB/NF-kB signaling in chondrocytes upon IL-1β exposure

**Figure 4.**
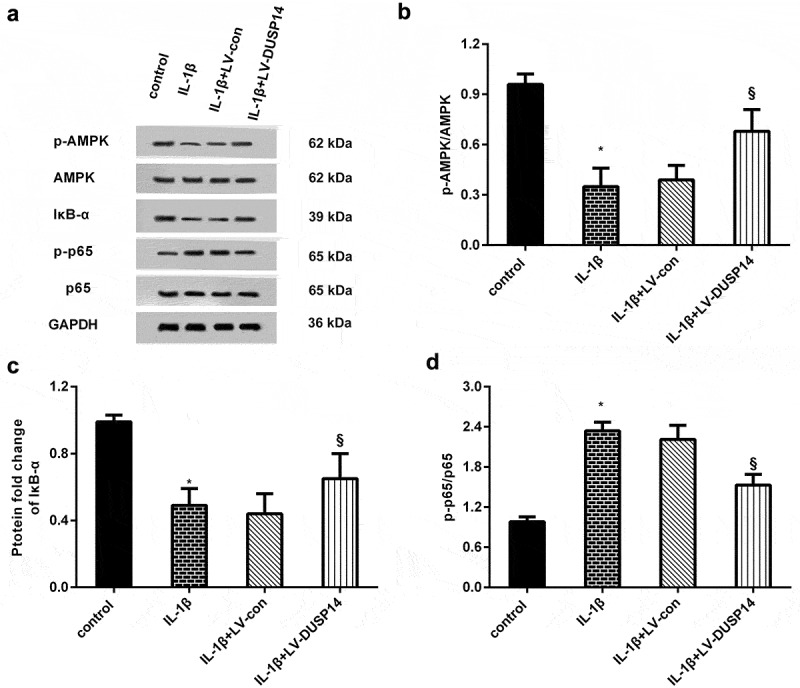
DUSP14 regulated the AMPK/IκB/NF-kB signaling in IL-1β-simulated chondrocytes. (a) After infection with LV-DUSP14 plasmids, cells were exposed to IL-1β. Then, western blotting was performed to determine the protein levels of p-AMPK, AMPK, IκB-α, p-p65 NF-κB and p65 NF-κB. (b-d) The corresponding protein bands were quantified by ImageJ software. n = 3. *P < 0.05 vs. control group. ^§^P < 0.05 vs. IL-1β-treated group

Accumulating evidence confirms the involvement of the AMPK/IκB/NF-kB signaling in inflammation-related diseases, including OA [21]. Therefore, we analyzed the correlation between DUSP14 and this pathway. As shown in Figure 4a-4d, the phosphorylation levels of AMPK (p-AMPK) were inhibited after IL-1β treatment, concomitant with a subsequent decrease in IκB-α expression and an increase in p-p65 NF-κB expression. Noticeably, DUSP14 enhancement elevated the protein expression of p-AMPK, IκB-α and suppressed p-p65 NF-κB levels in IL-1β-treated cells, indicating that DUSP14 overexpression may restrain the NF-κB signaling by activating the AMPK pathway.

## The AMPK/IκB/NF-kB signaling mediates chondroprotective efficacy of DUSP14 elevation

**Figure 5.**
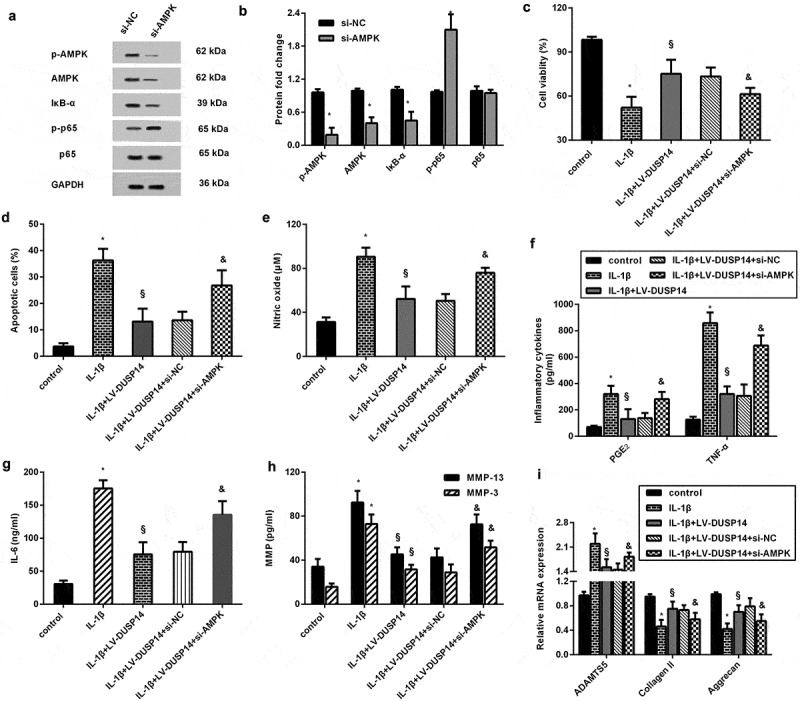
The AMPK/IκB/NF-kB signaling mediates chondroprotective efficacy of DUSP14 elevation. (a, b) After transfection with si-AMPK, the activation of the AMPK/IκB/NF-kB signaling was analyzed. n = 3. (c, d) Cells were treated with si-AMPK, LV-DUSP14 and IL-1β. Then, cell viability (c) and apoptosis (d) were determined. n = 4. (e-i) The subsequent efficacy on NO (e), PGE2 and TNF-α (f), IL-6 (g), MMP production (h) and other metabolism-related protein expression (i) were assessed. n = 3.*P < 0.05 vs. control group. ^§^P < 0.05 vs. IL-1β-treated group. ^&^P < 0.05 vs. IL-1β and LV-DUSP14 group

To clarify the involvement of AMPK/IκB/NF-kB signaling in DUSP14-mediated chondroprotection, si-AMPK was transfected into chondrocytes to block this pathway (Figure 5a and 5b). Functional assay demonstrated that DUSP14 elevation protected against IL-1β-induced chondrocyte injury by increasing cell viability (Figure 5c) and decreasing cell apoptosis (Figure 5d), whereas this effect was attenuated when AMPK signaling was blocked. Additionally, the suppressive effects of DUSP14 overexpression on IL-1β-evoked inflammatory levels were counteracted after silencing the AMPK pathway (Figure 5e-5g). Simultaneously, si-AMPK transfection also muted the regulatory function of DUSP14 elevation in metabolic dysfunction under IL-1β exposure by enhancing MMP-3, MMP-13, ADAMTS5 expression and decreasing collagen II and aggrecan expression (Figure 5h and 5i).

## Elevation of DUSP14 ameliorates the progression of OA *in vivo*

**Figure 6.**
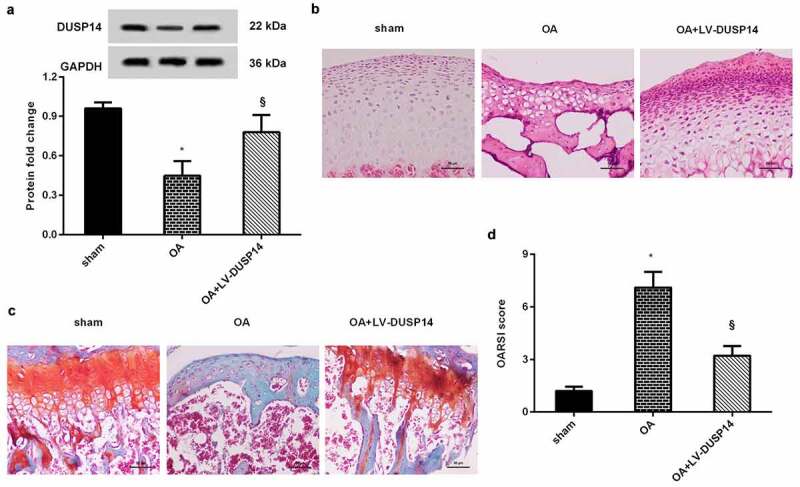
Elevation of DUSP14 alleviated OA progression *in vivo*. (a) Rats underwent ACLT were injected with LV-DUSP14 plasmids. Then, the protein expression of DUSP14 in articular cartilage was detected by western blotting. (b-d) Subsequently, histological assay was conducted by HE (b), Safranin O staining (c) and OARSI scores (d). n = 10 rats in each group. *P < 0.05 vs. sham group. ^§^P < 0.05 vs. OA group

We further elaborated *on* the function of DUSP14 in OA *in vivo*. As shown in Figure 6a, decreased protein levels of DUSP14 were observed in articular cartilage of ALCT-constructed OA rats relative to the sham groups. However, injection with the lentivirus vector LV-DUSP14 dramatically elevated DUSP14 expression in OA groups. Intriguingly, HE (Figure 6b) and Safranin O staining substantiated that compared with sham groups, OA groups displayed an obvious reduction in cartilage matrix and thickness, which were alleviated after DUSP14 overexpression. Additionally, injection with LV-DUSP14 also decreased OARSI scores relative to the OA groups (Figure 6d), implying the amelioration of cartilage degeneration in OA.

## Discussion

OA is known as a proverbial inflammatory degenerative joint disease with the acceleration of the aging process, and has troubled middle-aged and elderly people [22]. It is a well-known viewpoint that progressive cartilage degradation and chondrocyte dysfunction are the hallmarks of OA [7,9]. DUSP14 belongs to the DUSP family and plays a key role in multiple inflammatory diseases. For instance, DUSP14 protects against isoflurane-evoked inflammatory response and cognitive impairment in aged rats [14]. In the current study, we confirmed the down-regulation of DUSP14 in cartilage tissue of OA rats. Additionally, the decrease in DUSP14 expression was also validated in IL-1β-stimulated chondrocytes. Importantly, DUSP14 elevation attenuated cartilage damage in ACLT-induced OA rats. Thus, these findings suggest that DUSP14 may be a promising therapeutic strategy for OA.

Chondrocytes are the major effector cells in the articular cartilage. Normally, chondrocytes maintain the stability of ECM, and compromising chondrocyte function and survival may result in the failure of articular cartilage in OA [7]. Currently, chondrocyte injury is recognized as a dominant biological event associated with the pathogenic process of OA [7]. Inflammatory cytokine IL-1β is a key initial contributor to OA development and has been widely applied to stimulate chondrocytes to simulate OA *in vitro* [20,23]. Similar to previous findings [23], exposure to IL-1β inhibited chondrocyte viability and induced cell apoptosis. Intriguingly, in this study, DUSP14 overexpression antagonized IL-1β-induced chondrocyte injury by increasing cell viability and decreasing cell apoptosis. Analogously, DUSP14 may act as a potential therapeutic target to prevent ischemic stroke by attenuating cerebral apoptosis [12]. Strikingly, knockdown of DUSP14 accelerates cardiomyocyte apoptosis and subsequent cardiac injury progression [24]. Thus, these data indicate that DUSP14 may ameliorate the development of OA by attenuating chondrocyte injury.

It is increasingly evident that inflammation of joint usually plays a central role in mediating the pathogenic progression of OA [6,10]. Inflammatory components, such as inflammatory mediators and cytokines, can be induced by chondrocytes in joints of OA patients [25]. IL-1β is secreted in early OA and can further drive the inflammatory cascade and aggravate the development of OA [25]. Similar to prior data [20,26], stimulation with IL-1β induced chondrocytes to release NO, PGE2, IL-6 and TNF-α. Intriguingly, a substantial body of evidence supports a critical function of DUSP14 in inflammatory diseases. For instance, DUSP14 affords prominent neuroprotective intervention in aged rats by inhibiting the inflammatory response [14]. Additionally, DUSP14 suppresses inflammation-induced pathological alterations in liver [13]. Herein, DUSP14 enhancement antagonized IL-1β-induced inflammatory response in chondrocytes. Currently, targeting the inflammatory mechanism has been accepted as a promising approach to dampen OA [23,25]. Thus, DUSP14 may alleviate OA progression by blocking inflammation.

Extensive degeneration of the articular cartilage ECM is a key event in OA and is involved in the metabolic homeostasis between catabolism and anabolism [9]. It is known that articular cartilage ECM is composed majorly of collagen II and aggrecan, which are necessary for the biomechanical properties of cartilage. Normally, the anabolism of articular cartilage maintains a dynamic balance with catabolism evoked by MMPs. However, the occurrence of an inflammatory environment disturbs the metabolic homeostasis and induces aberrant catabolism by excessive production of aggrecanases and MMPs, such as MMP-3 and MMP-13 [27]. Accumulating findings confirm the abnormal levels of MMP-3 and MMP-13 in patients with OA [27,28]. Furthermore, the high production of MMPs may contribute to the degenerative processes during the progression of OA [27]. Intriguingly, this study found that elevation of DUSP14 overturned IL-1β-evoked catabolism and inhibition of anabolism. Therefore, DUSP14 may mediate IL-1β-induced metabolism disturbance toward catabolism, indicating a key role of DUSP14 in articular cartilage degeneration by regulating metabolic homeostasis between catabolism and anabolism.

Further elucidation of the mechanism corroborated that DUSP14 overexpression activated the AMPK signaling. AMPK is a principal participant in sustaining cellular energy homeostasis and has been implicated in multiple pathogenic processes, such as inflammation, metabolism and oxidative stress [21,29,30]. NF-κB signaling is a well-known inflammatory mechanism associated with various inflammatory diseases including OA [31]. The phosphorylation of AMPK is generally considered to suppress NF-κB activation by regulating IκBα, a key component of the IκB inhibitory family [29,31]. Currently, abundant evidence confirms the involvement of AMPK/NF-κB signaling in the development of OA [31,32]. Of note, the current data substantiated that DUSP14 elevation restored IL-1β-suppressed activation of AMPK signaling and subsequently inhibited the activation of the NF-κB pathway. Importantly, blockage of the AMPK pathway muted the protective efficacy of DUSP14 against IL-1β-induced chondrocyte injury, inflammation and ECM metabolism disturbance. Therefore, the activation of the AMPK signaling may account for the beneficial function of DUSP14 in IL-1β-evoked chondrocyte dysfunction.

## Conclusion

In summary, the current findings highlighted the down-regulation of DUSP14 in OA animal and cell models. Furthermore, DUSP14 overexpression alleviated cartilage degradation in OA rats, and antagonized IL-1β-induced chondrocyte inflammatory injury and metabolism disorders toward catabolism. The activation of the AMPK/IκB/NF-kB signaling was involved in above progression. Therefore, these data imply that DUSP14 may ameliorate the progression of OA by affecting chondrocyte injury, inflammation and metabolic homeostasis. Together, these data may provide an important theoretical basis for further investigation of DUSP14 in the clinical treatment of OA. Overall, DUSP14 may serve as a promising therapeutic approach against OA.

### Limitation/short coming of the study

Although this research provides some new clues to the function of DUSP14 in osteoarthritis progression, there are some deficiencies as well as limitations to this study. First, rat chondrocytes were used in our research, which could not completely mimic the characteristics of human chondrocytes. Second, the protective efficacy of DUSP14 was not completely reversed by the AMPK pathway. Whether there are other pathways involved in these processes. Third, we confirmed the protective efficacy of DUSP14 in OA rats. However, the similar results have not been validated in human experiments.

In the future, we need to investigate the function of DUSP14 in human chondrocytes under OA conditions. Furthermore, the roles of DUSP14 in diagnosis and prognosis of OA is worthy of further exploration. Finally, mechanical stress exerts the key roles in OA. Therefore, we will further elucidate the function of DUSP14 in mechanical load-induced OA progression.

## Data Availability

All data generated or analysed during this study are included in this published article.

## Significance of this study

Osteoarthritis (OA) is a common whole-point and debilitating disease worldwide and constitutes the major source of pain and disability among people. The current findings highlight that DUSP14 may ameliorate OA progression by affecting chondrocyte injury, inflammatory response and cartilage metabolism homeostasis. Therefore, this study may support DUSP14 as a promising therapeutic strategy against OA.

## Highlights

★The expression of DUSP14 was decreased in OA model *in vivo and in vitro*

★DUSP14 alleviated IL-1β-evoked chondrocyte injury and inflammatory response

★DUSP14 affects metabolic homeostasis in IL-1β-stimulated chondrocytes

★The AMPK/IκB/NF-kB signaling mediates chondroprotective efficacy of DUSP14 elevation

★Elevation of DUSP14 ameliorates the progression of OA *in vivo*
